# Hypersynchrony in sarcomeric hypertrophic cardiomyopathy: description and mechanistic approach using multimodal electro-mechanical non-invasive cartography (HSYNC study)

**DOI:** 10.3389/fcvm.2024.1359657

**Published:** 2024-06-07

**Authors:** Patricia Réant, Guillaume Bonnet, Frédérique Dubé, Charles Massie, Amélie Reynaud, Matthieu Michaud, Josselin Duchateau, Stéphane Lafitte

**Affiliations:** ^1^ Cardiology Department, Bordeaux University Hospital, Bordeaux, France; ^2^ Cardiology Department, University of Bordeaux, Bordeaux, France; ^3^ Cardiology Department, IHU Lyric, Bordeaux-Pessac, France; ^4^ Cardiology Department, CIC-P 1401, Bordeaux-Pessac, France; ^5^ Cardiology Department, INSERM 1045, Bordeaux, France; ^6^ Cardiology Department, Centre Hospitalier Universitaire de Sherbrooke, Sherbrooke, QC, Canada; ^7^ Cardiology Department, Sacred Heart Hospital of Montreal, Montreal, QC, Canada

**Keywords:** hypertrophic cardiomyopathy, non-invasive electrocardiographic mapping, cardiac activation, myocardial contraction, apex-to-base delay, hypersynchrony

## Abstract

**Background:**

Little is known about left ventricular (LV) sequences of contraction and electrical activation in hypertrophic cardiomyopathy (HCM). A better understanding of the underlying relation between mechanical and electrical activation may allow the identification of predictive response criteria to right ventricular DDD pacing in obstructive patients.

**Objective:**

To describe LV mechanical and electrical activation sequences in HCM patients compared to controls.

**Materials and methods:**

We prospectively studied, in 40 HCM patients (20 obstructive and 20 non-obstructive) and 20 healthy controls: (1) mechanical activation using echocardiography at rest and cardiac magnetic resonance imaging, (2) electrical activation using 3-dimensional electrocardiographic mapping (ECM).

**Results:**

In echocardiography, healthy controls had a physiological apex-to-base delay (ABD) during contraction (23.8 ± 16.2 ms). Among the 40 HCM patients, 18 HCM patients presented a loss of this ABD (<10 ms, defining hypersynchrony) more frequently than controls (45% vs. 5%, *p* = 0.017). These patients had a lower LV end-diastolic volume (71.4 ± 9.7 ml/m^2^ vs. 82.4 ± 14.8 ml/m^2^, *p* = 0.01), lower native T1 values (988 ± 32 ms vs. 1,028 ± 39 ms, *p* = 0.001) and tended to have lower LV mass (80.7 ± 23.7 g/m^2^ vs. 94.5 ± 25.3 g/m^2^, *p* = 0.08) compared with HCM patients that had a physiological contraction sequence. There was no significant relation between ABD and LV outflow tract obstruction. While HCM patients with a physiological contraction sequence presented an ECM close to those encountered in controls, patients with a loss of ABD presented a particular pattern of ECM with the first potential more frequently occurring in the postero-basal region.

**Conclusion:**

The LV contraction sequence can be modified in HCM patients, with a loss of the physiological ABD, and is associated with smaller LV dimensions and a particular pattern of ECM. Further research is needed to determine whether this pattern is related to an electrical substrate or is the consequence of the hypertrophied heart's specific geometry.

**Clinical trial registration:**

ClinicalTrial.gov: NCT02559726.

## Introduction

Hypertrophic cardiomyopathy (HCM) is the most common inherited cardiomyopathy, affecting 1/500 people in the general population (1). HCM can manifest as a varied range of phenotypic presentations marked by asymmetrical hypertrophy of the left ventricle (LV), irregularities of the mitral valve apparatus, and potential LV outflow tract obstruction (LVOTO). This LVOTO constitutes an important hallmark of HCM, with correlations to symptomatic expression, clinical outcomes, and the risk of sudden cardiac death (SCD) (2). The underlying pathophysiology of this obstruction is primarily associated with systolic anterior motion (SAM) of the mitral valve, resulting in contact between the mitral valve and the septal wall (3). Several mechanisms have been postulated to contribute to this phenomenon, including septal hypertrophy, heightened contractility (hyperkinesia), elongation of the anterior mitral valve, atypical positioning, and hypertrophy of the subvalvular apparatus. Furthermore, hemodynamic factors, notably pre-load conditions, have a significant impact on this process (4).

As per the guidelines (5–7), the primary interventions for addressing this obstruction involve the administration of negative inotropic agents (such as beta-adrenergic blockers, calcium channel blockers, disopyramide, and more recently, myosin inhibitors) as initial therapy. Invasive therapies include septal reduction through surgical myectomy or alcohol ablation, which are pursued in instances of persistent symptoms resistant to pharmacological treatment. Dual chamber pacing is also considered a potential therapeutic option. The concept of using dual chamber pacing with a permanent right ventricular pacemaker, in patients with HCM, has evolved throughout time. Its inception traces back to the 1960s and preliminary investigations during the early 1990s showcased encouraging outcomes (8, 9). However, randomized trials yielded conflicting results (10–12), relegating this approach to a secondary therapeutic option in guidelines. Nonetheless, more recent reports with extended follow-up periods have brought to light the clinical and hemodynamic advantages of pacing (13, 14).

To identify criteria predictive of a favorable response to pacing, it seems essential to analyze electrical and mechanical LV activation sequences. These reflect electrical depolarization and LV contraction, which follow a specific temporal rhythm, allowing synchronized ventricular contraction and progressive cavity emptying. The usual sequence of LV contraction introduces an element of asynchrony, as supported by the temporal disparities between early contraction of the apex and late contraction of the base (15). Within the scope of HCM, deviations from the established physiological asynchrony pattern have become evident (16). Specifically, a subgroup of patients exhibit hypersynchrony, which is a loss of the physiological apex-to-base delay (ABD), or inverted asynchrony, which is an inversion of this delay altogether.

We hypothesized that the presence of hypersynchrony could contribute to LVOTO and help predict the efficacy of right ventricular pacing. The HSYNC study was designed to achieve the following main objectives (1) describe LV mechanical and electrical activation sequences in HCM patients (2) compare these patterns to those of healthy controls and detect the presence of hypersynchrony (3) establish standardized measures through multimodal non-invasive electro-mechanical cartography.

## Materials and methods

### Study design, population

The HSYNC study is a monocentric observational cohort study including 20 patients with obstructive hypertrophic cardiomyopathy (O-HCM), 20 patients with non-obstructive hypertrophic cardiomyopathy (NO-HCM), and 20 healthy control subjects, all of which were analyzed through a prospective approach. Patient enrolment was done at the Hereditary Cardiomyopathy Reference Centre, part of Bordeaux University Hospital, France (NCT02559726). Eligible individuals were patients aged 18 years or older with a prior diagnosis of HCM based on consensus criteria (7) (non-dilated left ventricle with a maximal wall thickness ≥15 mm in the absence of comparable degrees of hypertrophy resulting from other cardiac or systemic conditions), and confirmed by genetic testing or family history. The presence of LVOTO was defined as per recommendations as an immediate peak Doppler gradient across the LV outflow tract of ≥30 mmHg at rest or with provocation (Valsalva maneuver and exercise). For the control group, eligibility was contingent on the absence of any cardiovascular pathology or risk factor.

Patients were excluded if they met any of the following criteria: pregnancy, presence of atrial arrhythmia, history of septal reduction therapy, insufficient ultrasound window for optimal imaging, apical form of hypertrophy, or any established contraindication to cardiac magnetic resonance imaging (CMR). Such contraindications included severe claustrophobia precluding CMR, hypersensitivity reactions to gadolinium-based contrast agents, and prior implantation of an intra-cardiac device incompatible with CMR procedures.

The study adhered to the principles outlined in the Declaration of Helsinki and received approval from the study center's Institutional Review Board or Independent Ethics Committee. The authors had unrestricted access to the study data and assume responsibility for the accuracy and reliability of this report.

During the in-hospital phase, a collection of data was gathered, including baseline characteristics, twelve-lead electrocardiograms, echocardiographic evaluations, and CMR parameters. The estimation of the risk for SCD was done using the HCM Risk-SCD formula (17). These comprehensive investigations were executed within a single day for each participant, and the protocols were as follows:

- For individuals affected with HCM:
  • Transthoracic echocardiography (TTE) assessments were conducted at rest and during a semi-supine bicycle test.
  • CMR procedures were performed and included the administration of gadolinium-based contrast agents for enhanced imaging.
  • Three-dimensional electrocardiographic mapping (ECM) exams were employed for precise electrocardiographic assessment.
- For control subjects:
  • TTE evaluations were performed at rest only.
  • CMR examinations were conducted without the administration of gadolinium-based contrast agents.
  • Three-dimensional ECM allowed detailed electrocardiographic evaluations.

### Echocardiography assessment

Resting and exercise TTE examinations were conducted using a Vivid E95 ultrasound machine (General Electric Medical System, Horten, Norway). Ultrasound recordings were captured in standardized views, using 2-dimensional imaging with frame rates exceeding 50 Hz. The modalities employed included pulsed, continuous, and color Doppler, with the recorded data being stored for centralized analysis. Characterization of hypertrophy followed the classification established by Maron (18). Particular emphasis was placed on the LVOT area to assess for SAM of the mitral valve. Continuous Doppler scans were conducted in this area to quantify the maximal outflow velocity. Pulsed wave tissue Doppler imaging analysis was done in the apical four-chamber view, focusing on the lateral and septal portions of the mitral annulus, as well as the lateral portion of the tricuspid annulus. Furthermore, the quantification of LV peak systolic global longitudinal strain was done using the two-dimensional speckle tracking method, employing a 17-segment model, and was performed across the three apical views.

Exercise TTE was conducted while maintaining the patient's medication regimen, adhering to the guidelines outlined by the European Association of Echocardiography (19). The exercise protocol involved bicycle exertion in a semi-supine position (50°), slightly tilted to the left. The workload was initiated at 30 Watts and increased by 30 Watts every 2 min until the maximum tolerable effort was reached. Throughout the exercise stages, from rest to recovery, conventional recordings were acquired, including the quantification of LVOTO. In addition to continuous electrocardiogram monitoring, systolic and diastolic blood pressures were measured during each stage of exercise. Inadequate blood pressure response to exercise was defined as a failure to increase systolic blood pressure by a minimum of 20 mmHg or a decline in systolic blood pressure at peak effort of ≥15 mmHg.

### Cardiac magnetic resonance imaging

All participants underwent CMR imaging with a 1.5 Tesla scanner (Magnetom Avanto, Siemens Medical Systems, Erlangen, Germany). The CMR scans included a series of localized images (axial, coronal, and sagittal planes), cine scans, native T1 mapping, and, exclusively for patients with HCM, late gadolinium enhancement (LGE) imaging, as well as post-contrast T1 mapping. Following localization, cine images of the LV were acquired during breath-hold using retrospectively gated sequence in various views, including 4-, 3-, and 2-chamber apical views, as well as short-axis views. The short-axis cine images were segmented to calculate LV volumes, mass, and ejection fraction, using the modified Simpson's rule. LGE imaging was captured 10–12 min after administration of a gadoterate meglumine contrast agent (0.2 mmol/kg) to visualize the entire LV. A qualitative analysis was applied to assess LGE patterns.

For T1 mapping, images were acquired both before and after the administration of the contrast agent, utilizing the modified Look Locker inversion-recovery (MOLLI) sequence. To derive myocardial T1 values, regions of interest were manually delineated in the septal region at the mid-ventricular level, avoiding regions of LGE, in both pre-contrast and post-contrast T1 maps. T1 value was obtained by placing a circular region of interest in the LV cavity while the calculation of myocardial extracellular volume (ECV) was done according to established methods (20).

### Velocity vector imaging and phase analysis

The assessment of contraction sequences was conducted using the Syngo Velocity Vector Imaging (VVI) software (Siemens Medical, Erlangen, Germany). This technique offers an angle-independent analysis of movement, velocity, strain, and strain rate. It employs an algorithm that integrates speckle tracking, tissue-blood border detection, mitral annulus motion, and cardiac periodicity analysis for endocardial tracking. The analyses were done from two perspectives: (1) From TTE loops in an apical 4-chamber view: coordinates were extracted for each point at each time frame, facilitating the analysis of diameter variations, which are presumed to be a result of circumferential strain. (2) From CMR loops in two short-axis views—one at the base (above mitral valve leaflets) and the other at the apex (downstream of the insertion of papillary muscles): variations in circumferential strain were analyzed at both levels. Using both TTE and CMR data to characterize contraction sequences will allow us to study the feasibility and reproducibility of each modality and increase the robustness of our findings.

The XLstat software (Addinsoft, Paris, France) was used to quantify the phase delay associated with the apex-base delay (ABD). This delay is characterized as the temporal discrepancy in milliseconds between the apical and basal points. A positive value denotes that the apex contracts before the base, while a negative value signifies that the base's contraction precedes that of the apex.

### Three-dimensional electrocardiographic mapping

Ventricular epicardial activation maps were obtained using a non-invasive, high-resolution ECM (CardioInsight Technologies Inc., Cleveland, Ohio), during the heart's intrinsic rhythm (21). The procedure involved 250 electrodes recording body surface potentials on strips distributed around the entire torso and a thoracic non-contrast CT scan. Each electrode featured a marker detectable on CT. By combining the body surface potentials and CT images, it became possible to reconstruct epicardial potentials, as well as activation and repolarization maps. Qualitative analysis of the activation maps was carried out, wherein the initial breakthrough site on the epicardium was identified as the earliest location of activation. Conversely, the region that activated last was also identified. From these activation maps, various electrical parameters were extracted: (1) total ventricular (right and left) activation time defined as the duration from the earliest to the latest point of ventricular activation; (2) total left ventricular activation time (3) ventricular electrical uncoupling defined as the mean left and right ventricular activation times (positive value indicates activation originating from the right ventricle, whereas a negative value indicates activation originating from the left ventricle); (4) ABD defined as the time difference between the apical point and the inferior basal point, which is typically the last to activate in a normal sequence of electrical activation.

### Statistical analyses

Baseline characteristics are summarized as means and standard deviations or medians with interquartile range for continuous measures and frequencies with proportions for categorical variables. Between the study groups, variables were compared with the analysis of variance (ANOVA) or the non-parametric Kruskal-Wallis test (in cases where the explanatory variables did not meet the assumptions of normality and variance homogeneity) for continuous metrics, and the chi-squared (*χ*2) test or the Fisher exact test for categorical variables. To examine the correlation between continuous variables, the rank-based Kendall's tau coefficient and Bland-Altman analysis were used. The evaluation of inter- and intra-observer variabilities involved calculating Pearson's correlation coefficient. Statistical analyses were performed using R (version 2023.03.0 + 386, R Foundation for Statistical Computing).

To assess intra- and inter-observer variability for ABD determined with TTE and CMR, cases (both controls and HCM patients) were reanalyzed off-line by the original observer and by a second observer, who was blinded to the initial results. We evaluated intra-observer variability and inter-observer variability in the determination of ABD, using both TTE and CMR imaging techniques.

## Results

### Baseline characteristics

Sixty patients were enrolled, including 20 patients with NO-HCM, 20 patients with O-HCM and 20 controls (Tables 1, 2). The mean age of patients with HCM was 53 ± 16 years, with 26 (65%) being male. There were no statistically significant differences in age and gender distribution between the patients and the control group. Compared to control subjects, HCM patients exhibited statistically higher values for LV maximal wall thickness, LV mass, and left atrial (LA) volume, while displaying a lower LV end-diastolic diameter.

**Table 1:** Baseline clinical characteristics.

	Controls	NO-HCM	O-HCM
	(*n* = 20)	(*n* = 20)	(*n* = 20)
Demographics			
Age, years	47 ± 16	50 ± 18	55 ± 13
Male	11 (55)	13 (65)	13 (65)
Body surface area, m^2^	1.8 ± 0.2	1.8 ± 0.2	2.0 ± 0.2
Heart rate, beats/min	68 ± 13	62 ± 13	63 ± 10
NYHA functional class			
I	20 (100)	10 (50)	4 (20)
II	0 (0)	7 (35)	10 (50)
III	0 (0)	3 (15)	6 (30)
Age at diagnosis, years	N/A	37 ± 19^‡^	50 ± 18
Family history of HCM	N/A	9 (45)	6 (30)
Diabetes mellitus	0 (0)	0 (0)	1 (5)
Hypertension	0 (0)	5 (25)^‡^	13 (65)
Paroxysmal atrial fibrillation	0 (0)	7 (35)^‡^	1 (5)
Sudden cardiac death risk score, %	0 (0)	2.3 ± 1.2	2.4 ± 1.6
Genetic			
Mutation identified	-	5 (25)	7 (35)
MYH7	-	2 (10)	4 (20)
MYBPC3	-	3 (15)	3 (15)
Medications			
Beta-blocker	0 (0)	10 (50)	14 (70)
Calcium channel blocker	0 (0)	3 (15)	2 (10)
Disopyramide	0 (0)	0 (0)	1 (5)
Amiodarone	0 (0)	1 (5)	1 (5)
Sotalol	0 (0)	4 (20)	0 (0)
ACE inhibitor/ARB	0 (0)	4 (20)	5 (25)
Diuretics	0 (0)	3 (15)	3 (15)
Oral anticoagulant	0 (0)	8 (40)	2 (10)

Values are mean ± standard deviation (*n*) or % (*n/N*).

ACE, Angiotensin II converting enzyme; ARB, angiotensin II receptor blocker; HCM, hypertrophic cardiomyopathy; NO-HCM, non-obstructive HCM; MYPBC3, myosin binding protein C3; MYH7, myosin heavy chain 7; NYHA, New York Heart Association; O-HCM, obstructive HCM.

**Table 2:** Admission and procedural characteristics.

	Controls	NO-HCM	O-HCM
	(*n* = 20)	(*n* = 20)	(*n* = 20)
Echocardiography			
LV maximal wall thickness, mm	8.2 ± 1.4*	20.2 ± 5.6	17.1 ± 3.4
Indexed LV end-diastolic diameter, mm/m^2^	26.1 ± 2.2*	23.6 ± 3.4	23.2 ± 2.4
LV ejection fraction, %	66.5 ± 5.8	67.6 ± 8.1	70.5 ± 7.2
LV global longitudinal strain, %	−22.0 ± 2.2***	−19.1 ± 4.3	−20.3 ± 4.0
Resting LVOT gradient, mmHg	-	8 ± 7**	70 ± 50
Maron class type			
I	-	1 (5)	5 (25)
II	-	16 (80)	13 (65)
III	-	3 (15)	2 (10)
Mitral regurgitation grade	**	**	
None or trivial	20 (100)	19 (95)	13 (65)
Mild	0 (0)	1 (5)	4 (20)
Moderate	0 (0)	0 (0)	3 (15)
Severe	0 (0)	0 (0)	0 (0)
SAM	N/A	3 (15)**	15 (75)
Indexed LA volume, ml/m^2^	23.3 ± 4.0*	36.4 ± 8.3**	45.9 ± 15.2
E/A ratio	1.3 ± 0.4	1.3 ± 0.7	1.0 ± 0.4
Mean E/e′ ratio	6.3 ± 1.4**	9.4 ± 4.0	10.3 ± 3.8
Systolic pulmonary artery pressure, mmHg	24.1 ± 4.6	29.2 ± 10.7	30.6 ± 7.6
S’TDI tricuspid annulus, cm/s	14.2 ± 1.8	13.2 ± 2.2	14.4 ± 3.3
Cardiac magnetic resonance imaging			
Indexed LV end-diastolic volume, ml/m^2^	73.1 ± 10.2	77.7 ± 15.4	77.0 ± 16.9
LV ejection fraction, %	63.2 ± 5.7	67.5 ± 8.9	70.9 ± 8.0
Indexed LV mass, g/m^2^	56.0 ± 9.1*	91.9 ± 26.9	84.3 ± 27.4
LGE	0 (0)	15 (75)	11 (55)
Native T1, ms	980 ± 27*	1,016 ± 49	1,005 ± 32
Extracellular volume	N/A	0.27 ± 0.04	0.24 ± 0.03

Values are mean ± standard deviation, median [Q1, Q3] or % (*n/N*).

LA, left atrium; LGE, late gadolinium enhancement; LV, left ventricle; LVOT, LV outflow tract; SAM, systolic anterior movement (of the mitral valve); TDI, tissue Doppler imaging.

* *p* < 0.05 vs. all HCM.

** *p* < 0.05 vs. O-HCM.

*** *p* < 0.05 vs. NO-HCM.

Compared to patients with O-HCM, patients with NO-HCM were diagnosed at a younger age, had a higher prevalence of paroxysmal atrial fibrillation, a lower incidence of hypertension, and a smaller LA volume. There was also a trend towards higher LV maximal wall thickness in NO-HCM patients (*p* = 0.09). The maximal LV outflow tract gradient at rest was measured to be 48 ± 43 mmHg (median 38 mmHg, IQR 14 to 58 mmHg) in O-HCM patients, in contrast to 6 ± 5 mmHg in NO-HCM patients (*p*-value < 0.0001). Among the O-HCM patients, LV obstruction was observed at rest in 11 patients (55%), after the Valsalva maneuver in 16 patients (80%), and during exercise in 17 patients (85%).

Exercise TTE results for HCM patients are detailed in Table 3. In comparison to O-HCM patients, NO-HCM patients exhibited a lower severity of mitral regurgitation and a tendency towards a less abnormal blood pressure response to exercise (*p*-value = 0.19). No significant difference was observed in terms of exercise capacity.

**Table 3:** Stress transthoracic echocardiography characteristics for HCM patients.

	NO-HCM	O-HCM
	(*n* = 20)	(*n* = 20)
Maximal charge, Watts	124 ± 37	118 ± 34
Total exercise time, min	8.3 ± 2.3	7.8 ± 2.0
Percentage of calculated maximal heart rate, %	76 ± 11	77 ± 13
Peak systolic BP, mmHg	166 ± 23	168 ± 38
Abnormal BP response to exercise	3 (15)	7 (35)
Exercise LVOT gradient, mmHg	13 ± 9	62 ± 46*
Mitral regurgitation grade during exercise		*
None or trivial	19 (95)	11 (55)
Mild	1 (5)	4 (20)
Moderate	0 (0)	4 (20)
Severe	0 (0)	1 (5)
SAM during exercise	3 (15)	15 (75)*

Values are mean ± standard deviation, median [Q1, Q3] or % (*n/N*).

BP, blood pressure; HCM, hypertrophic cardiomyopathy; LV, left ventricle; LVOT, LV outflow tract; NO-HCM, non-obstructive HCM; O-HCM, obstructive HCM; SAM, systolic anterior movement (of the mitral valve).

* *p* < 0.05 between the two HCM subpopulations.

### Left ventricular contraction sequence

**Figure 1:**
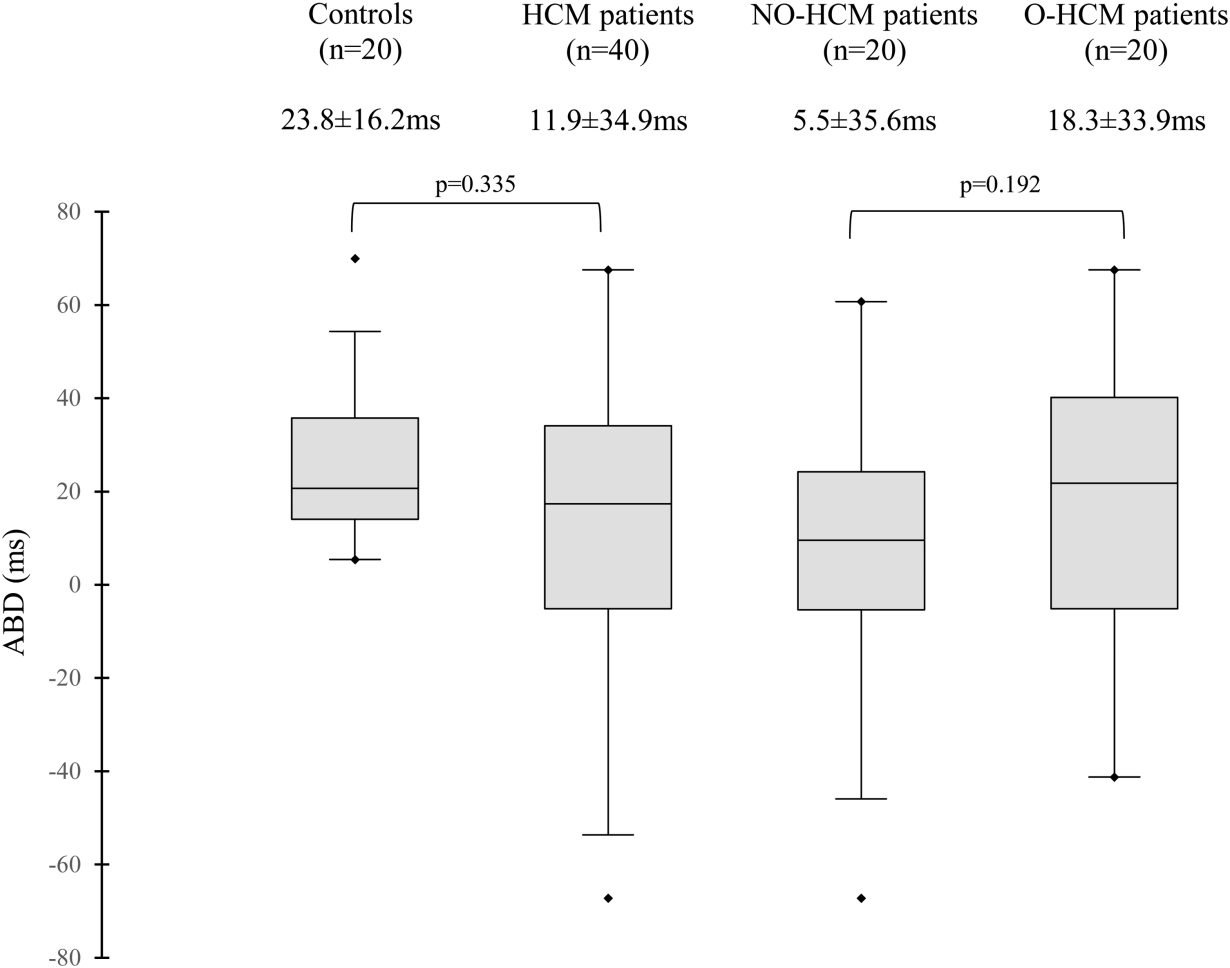
Distribution of apex-to-base delay in left ventricular contraction in the different subgroups. ABD, apex-to-base delay; HCM, hypertrophic cardiomyopathy; NO-HCM, non-obstructive HCM; O-HCM, obstructive HCM. ABD is determined with transthoracic echocardiography and corresponds to phase (*φ*) difference between apical and basal points (*φ* base—*φ* apex), a positive value reflects a contraction of apex before base, and a negative value a contraction of base before apex.

**Figure 2:**
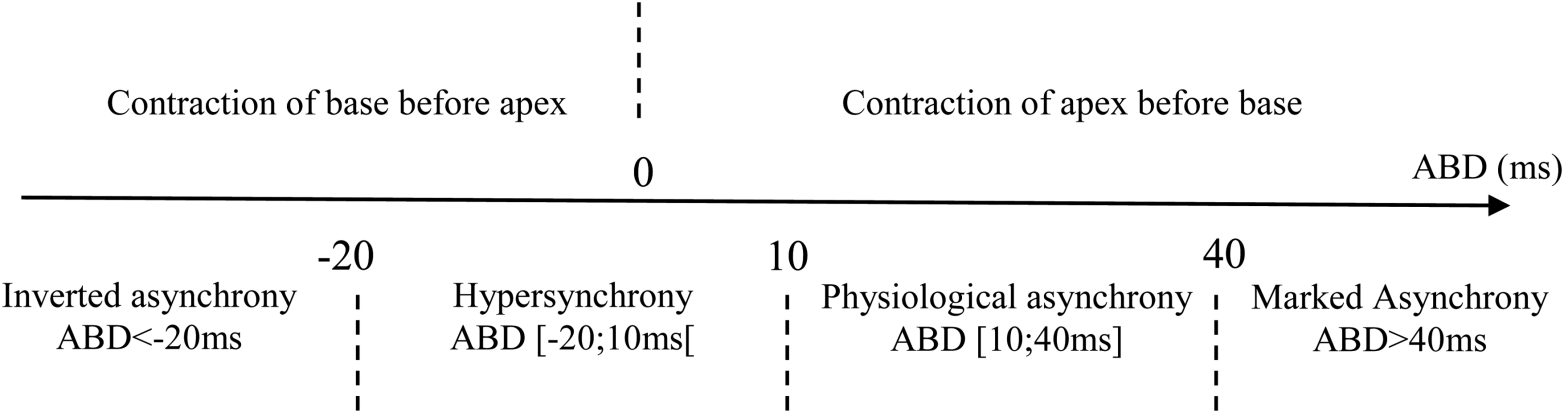
Definitions of contraction sequence profiles according to apex-to-base delay (ABD).

An analysis of contraction sequences in control subjects revealed a delay between the contraction of the apex and the base, with a mean ABD of 23.8 ± 16.2 ms, and a median of 20.7 ms [IQR (14.0–35.8 ms)], as shown in Figure 1. Based on these findings, we defined four contraction sequence profiles (Figure 2):

1. Physiological asynchrony: ABD between 10 and 40 ms (observed in 70% of controls).
2. Marked asynchrony: ABD >40 ms (observed in 20% of controls).
3. Hypersynchrony: ABD between −20 and 10 ms (observed in 10% of controls).
4. Inverted asynchrony: ABD < −20 ms (not observed in controls).

The mean ABD in the entire HCM population was 11.9 ± 34.9 ms, with a median of 17.4 ms [IQR (−5–34 ms)], which was not significantly reduced compared to controls (*p* = 0.34). There was no statistical difference between patients with NO-HCM and O-HCM (*p* = 0.192). The distribution of ABD between these groups is presented in Figure 1. This delay was not influenced by age (*τ* = 0.09, *p* = 0.38) or treatments that slow electrical conduction, such as beta-blockers, calcium channel blockers, and disopyramide (*p* = 0.754).

**Figure 3:**
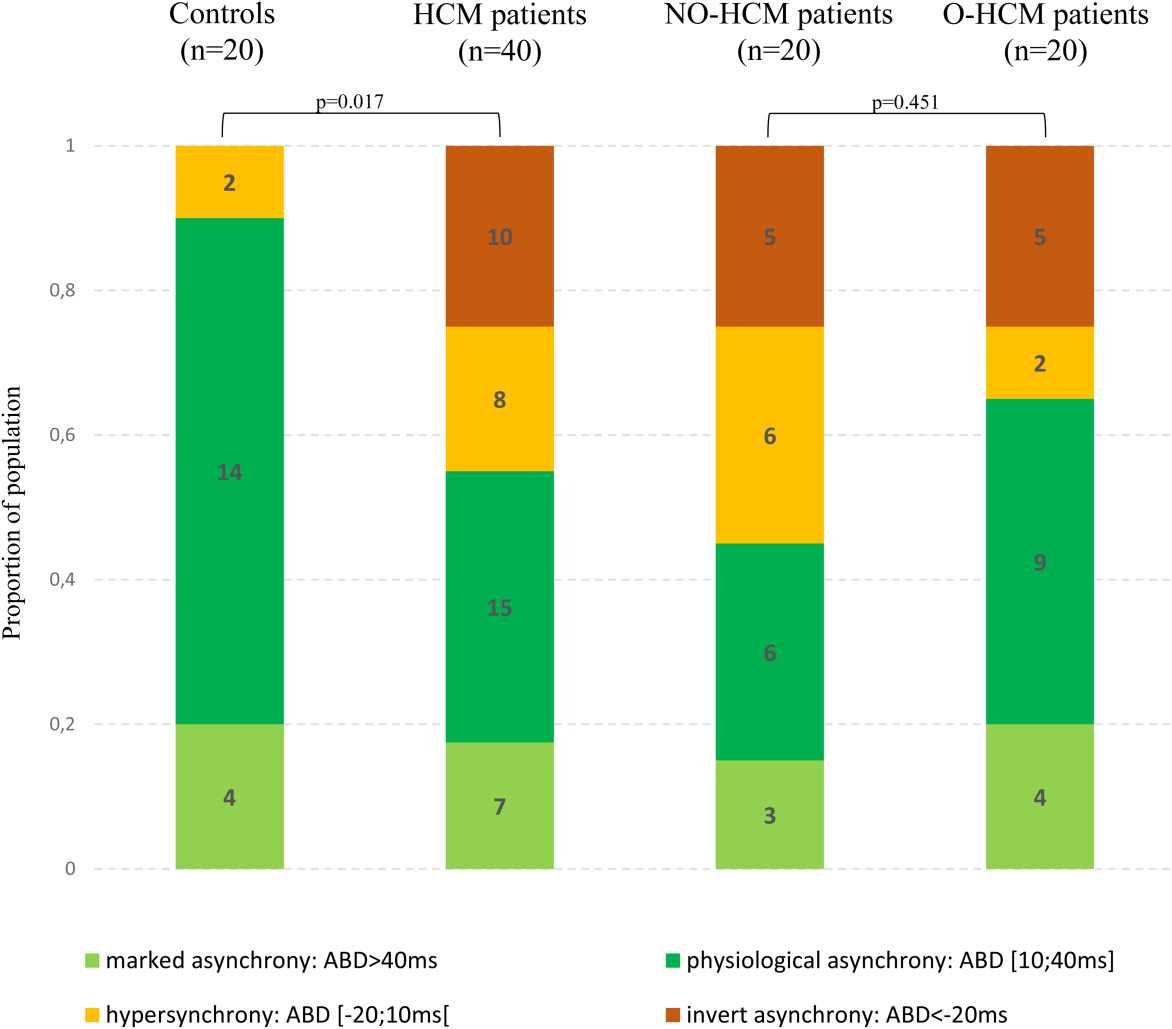
Distribution of contraction sequence profiles in the different subgroups.

Among HCM patients, 18% exhibited marked asynchrony, 38% had physiological asynchrony, 20% displayed hypersynchrony, and 25% showed inverted asynchrony. Consequently, among the 40 HCM patients, 45% experienced a loss of the delay between apical and basal contraction (hypersynchrony or inverted asynchrony), while it was preserved in 55% of them (physiological asynchrony or marked asynchrony). The distribution of contraction sequence profiles between groups is presented in Figure 3. Profiles of contraction with a loss of physiological ABD were significantly more prevalent in HCM patients compared to controls (*p* = 0.017). There was no difference in the distribution of contraction sequence profiles between obstructive and non-obstructive HCM patients (*p* = 0.451).

**Figure 4:**
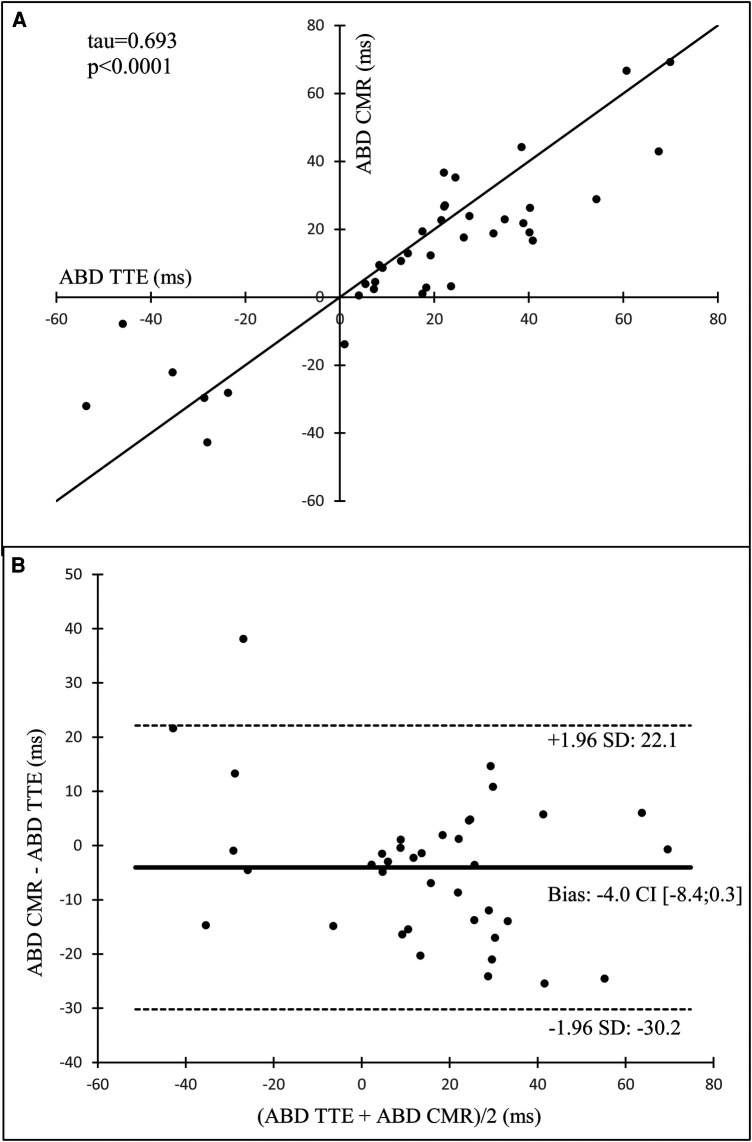
Kendall's correlation (**A**) and Bland-Altman analysis (**B**) of apex-to-base delay in left ventricular contraction determined with transthoracic echocardiography (ABD TTE) versus those determined with cardiac magnetic resonance imaging (ABD CMR). HCM, hypertrophic cardiomyopathy; NO-HCM, non-obstructive HCM; O-HCM, obstructive HCM. Apex-to-base delay (ABD) is determined with transthoracic echocardiography and corresponds to phase (*φ*) difference between apical and basal points (*φ* base—*φ* apex), a positive value reflects a contraction of apex before base, and a negative value a contraction of base before apex.

Analysis of CMR loops allowed for the characterization of contraction sequence profiles and ABD for 38 subjects (13 control subjects and 25 HCM patients, including 12 obstructive and 13 non-obstructive). A good correlation was observed between ABD obtained by both modalities (*τ* = 0.693, *p* < 0.0001), as shown in Figure 4.

### Comparison of the different LV contraction sequence subpopulations

The HCM population was divided into two subpopulations based on LV contraction sequences as determined with TTE: patients with preserved ABD (physiological asynchrony or marked asynchrony) and patients with a loss of ABD (hypersynchrony or inverted asynchrony). A comparison of these subpopulations of HCM patients is presented in Table 4. Patients with a loss of ABD, as compared to those with preserved ABD, exhibited a lower LV end-diastolic volume index (71.4 ± 9.7 ml/m^2^ vs. 82.4 ± 14.8 ml/m^2^, *p* = 0.01) and a lower native T1 (988 ± 32 ms vs. 1,028 ± 39 ms, *p* = 0.001). They also tended to have a lower LV mass index (80.7 ± 23.7 g/m^2^ vs. 94.5 ± 25.3 g/m^2^, *p* = 0.08) and a higher E/A ratio (1.34 ± 0.76 vs. 1.03 ± 0.43, *p* = 0.11). The proportion of males was lower in patients with hypersynchrony or inverted asynchrony compared to patients with physiological or marked asynchrony (50% vs. 77%), although this difference was not statistically significant (*p* = 0.08). No differences were observed between contraction sequence profiles in terms of functional class, exercise capacity, peak gradient, LV ejection fraction, LV global longitudinal strain, septal wall thickness, mitral regurgitation, or SAM. Within the HCM population, there was no significant correlation between ABD and maximal peak gradient (*τ* = −0.07, *p* = 0.58) or septal wall thickness (*τ *= 0.10, *p* = 0.41).

**Table 4:** Comparison of HCM subpopulations.

	Hypersynchrony or inverted asynchrony	Physiological asynchrony or marked asynchrony	*p*-value
	(*n* = 18)	(*n* = 22)	
Demographics			
Age, years	51 ± 18	54 ± 15	0.88
Male	9 (50)	17 (77)	0.08
SCD risk score, %	2.3 ± 1.2	2.4 ± 1.5	0.97
NYHA functional class			0.45
I	7 (38)	7 (32)	
II	8 (44)	9 (41)	_ _
III	3 (18)	6 (27)	
Maximal charge, Watts	123 ± 38	119 ± 34	0.32
Total exercise time, min	8.5 ± 2.0	7.7 ± 2.0	0.22
Peak systolic BP, mmHg	170 ± 35	165 ± 27	0.56
Abnormal BP response to exercise	3 (18)	8 (36)	0.18
Echocardiography			
O-HCM/NO-HCM	7/11	13/9	0.33
Maximal LVOT gradient, mmHg	82.3 ± 18.8	95.8 ± 46.5	0.32
	24 [15–79]	42 [21–89]	
Indexed LV end-diastolic diameter, mm/m^2^	22.8 ± 2.8	23.8 ± 3.0	0.29
LV maximal wall thickness, mm	19.1 ± 5.2	18.3 ± 4.7	0.58
Biplane LV Ejection fraction, %	68.7 ± 8.7	69.3 ± 6.9	0.82
LV global longitudinal strain, %	−19.5 ± 4.3	−19.8 ± 4.1	0.80
Mitral regurgitation grade			0.29
None or trivial	16 (88)	16 (73)	
Mild	1 (6)	4 (18)	
Moderate	1 (6)	2 (9)	
Severe	0 (0)	0 (0)	
SAM	6 (33)	11 (50)	0.45
Indexed LA volume, ml/m^2^	38.8 ± 10.4	43.0 ± 14.9	0.53
E/A ratio	1.34 ± 0.76	1.03 ± 0.43	0.11
ABD, ms	−18.1 ± 25.4	36.5 ± 18.5	<0.0001
Cardiac magnetic resonance imaging			
Indexed LV end-diastolic volume, ml/m^2^	71.4 ± 9.7	82.4 ± 14.8	0.01
Indexed LV mass, g/m^2^	80.7 ± 23.7	94.5 ± 25.3	0.08
LGE	13 (72)	13 (59)	0.37
Native T1, ms	988 ± 32	1,028 ± 39	0.001
Extracellular volume	0.25 ± 0.03	0.26 ± 0.03	0.88

Values are mean ± standard deviation, median [Q1, Q3] or % (*n*/*N*).

ABD, apex to base delay; BP, blood pressure; LA, left atrium; LGE, late gadolinium enhancement; LV, left ventricular; LVOT, LV outflow tract; NO-HCM, non-obstructive HCM; NYHA, New York Heart Association; O-HCM, obstructive HCM SAM, systolic anterior movement; SCD, sudden cardiac death.

* *p* < 0.05 between the two HCM subpopulations.

### Electrical activation sequence and correlation with contraction sequence

The mean QRS duration among HCM patients was 101 ± 19 ms. Among them, 2 patients (5%) exhibited a complete left bundle branch block, 2 patients (5%) had a complete right bundle branch block, and 1 patient (2.5%) showed a non-specific interventricular conduction delay. Notably, there was a modest correlation observed between ABD and QRS duration (*τ *= 0.291, *p* = 0.01) (Table 5).

**Table 5:** Correlation between apex-to-base delay in left ventricular contraction and electrical activation parameters obtained from electrocardiogram and electrocardiographic mapping in hypertrophic cardiomyopathy population.

		Tau	Confidence interval	*p* value
Electrocardiogram	QRS duration, ms	0.291	[0.083; 0.474]	0.01
Electrocardiographic mapping	VTAT, ms	0.217	[−0.012; 0.425]	0.07
	LVTAT, ms	0.145	[−0.086; 0.362]	0.23
	VEU, ms	−0.144	[−0.349; 0.074]	0.21
	ABD, ms	0.508	[0.312; 0.662]	<0.0001

ABD, apex to base delay; LVTAT, left ventricular total activation time; VEU, ventricular electrical uncoupling; VTAT, ventricular total activation time.

Electrocardiographic mapping data were available for 34 HCM patients, comprising 15 patients with hypersynchrony or inverted asynchrony and 19 patients with physiological asynchrony or marked asynchrony. Analysis of activation maps revealed that in HCM patients with preserved ABD, the initial epicardial breakthrough predominantly occurred in the right para-septal region (in 68% of cases) and infrequently in the postero-basal region (10% of cases). Conversely, in patients exhibiting a loss of physiological ABD, this distribution was reversed, with the first potential occurring mainly in the postero-basal region (in 47% of cases) and less frequently in the para-septal region (in 33% of cases vs. 68% in HCM patients with preserved ABD). This pattern was particularly pronounced in patients with inverted asynchrony, where 7 out of 9 patients (78%) with this contraction sequence profile exhibited an activation beginning in the postero-basal region.

**Figure 5:**
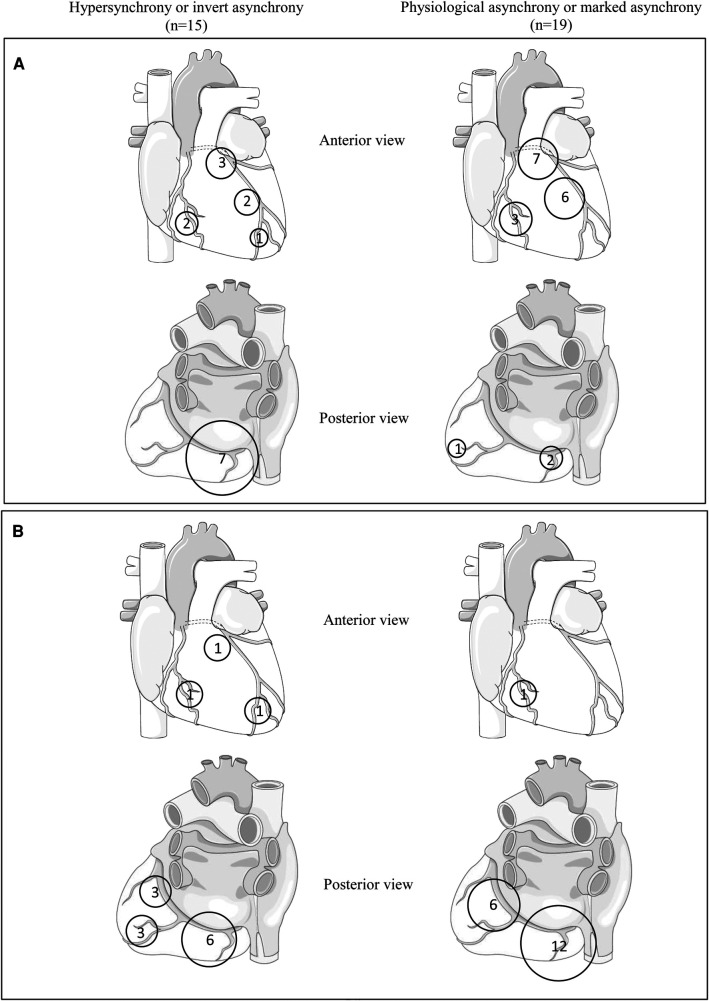
Summary of first (**A**) and last (**B**) regions to activate on electrocardiographic mapping obtained from the hypertrophic cardiomyopathy population.

**Figure 6:**
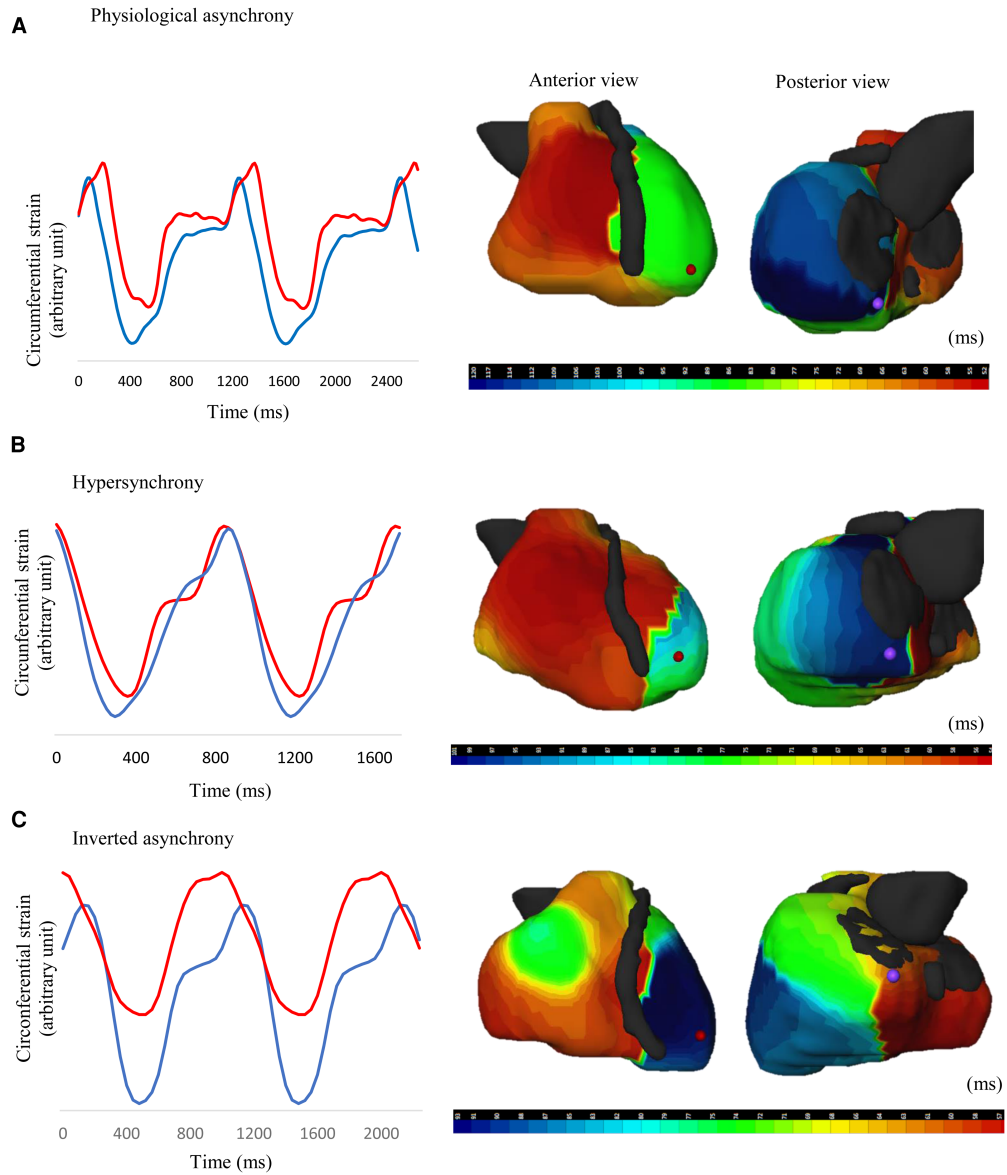
Comparative analysis of different LV contraction and activation profiles in patients with HCM. (**A**) Non-obstructive HCM patient exhibiting physiological asynchrony [apex-to-base delay (ABD) = 57 ms] (**B**) Non-obstructive HCM patient exhibiting hypersynchrony (ABD = 9 ms) (**C**) Obstructive HCM patient exhibiting inverted asynchrony (ABD = −41 ms). Apical motion is represented by the *blue line*; basal motion is represented by the *red line*.

An analysis of the location of the last region to activate demonstrated that in HCM patients with preserved ABD, this zone was almost exclusively situated in the basal region of the LV, either anterior or posterior, in 95% of cases. However, there was a higher variability in the subgroup of patients with a loss of ABD. The locations of the first and last regions to activate are summarized in Figure 5. A moderate correlation was observed between ABD in LV contraction and LV activation (*τ* = 0.508, *p* < 0.0001), but no correlation was found with other indices derived from electrocardiographic mapping, such as total ventricular activation time, total left ventricular activation time, and ventricular electrical uncoupling (detailed in Table 5). Examples of different contraction sequence profiles are provided in Figure 6, along with comparative analyses of motion obtained from Vector Velocity Imaging (VVI) and electrocardiographic mapping (ECM).

The intra-observer and inter-observer correlation coefficients for ABD were 0.80 and 0.72 respectively, when measured with TTE, and 0.85 and 0.77 respectively, when measured with CMR.

## Discussion

This study was the first to comprehensively examine a subgroup of HCM patients with an abnormal LV contraction sequence, characterized by loss of the physiological apex-to-base delay in LV contraction. Patients with this contraction profile exhibited lower LV volumes and native T1 values. They also tended to be predominantly female and to have a lower LV mass. Additionally, these patients showed distinctive patterns on electrocardiographic mapping. We confirmed the feasibility and reproducibility of assessing the apex-to-base delay using VVI on TTE and CMR images. We found a strong correlation between ABD obtained via TTE and CMR (*τ *= 0.69; *p* < 0.0001).

### Analysis phase

In our analysis of TTE loops, we opted for 2-dimensional TTE over 3-dimensional TTE due to superior image quality and frame rate, which is crucial to obtain the temporal resolution required for delay analysis. We selected LV circumferential strain over longitudinal strain because there have been reports indicating the potential reduction of global longitudinal strain in HCM, with spatial heterogeneity in these alterations (22). Furthermore, circumferential strain plays a significant role in systolic ejection (23). We favored the 4-chamber view over the parasternal short-axis view because it allows for simultaneous visualization of apex and base segments and overcomes alignment difficulties often encountered with short-axis views. However, the 4-chamber view offers only a 2-dimensional approach, necessitating validation against CMR short-axis views.

### Contraction sequence

Previous studies have described a physiological delay in the apex-to-base contraction sequence in the left ventricle (16). Utilizing velocity vector imaging and phase analysis, we were able to confirm a delay in circumferential contraction (averaging 24 ms) in control subjects. The loss of this physiological delay in HCM patients was recently reported. Our study confirmed the presence of this abnormal contraction sequence in a subset of HCM patients (45% of our study population), characterized by simultaneous contraction (hypersynchrony) or an inverted contraction sequence (inverted asynchrony). This loss of ABD was associated with a distinct LV geometry: a smaller LV cavity (lower end-diastolic volume and mass) resulting in a higher relative septal thickness. This can also explain why females tended to be more affected than males, as they are known to have smaller LV cavities with similar levels of septal thickness and thus a higher degree of relative hypertrophy. While T1 values were significantly increased in our HCM patients compared to controls, loss of ABD was associated with lower T1 values pointing to less myocardial fibrosis. This suggests that this particular pattern of mechanical activation is not mediated by increased myocardial scarring and more advanced disease, but by alternative mechanisms. It probably corresponds to an early phenomenon in the natural history of HCM, as is myocardial hypercontractility.

### Electrical activation sequence

There is limited data available on the electrical activation sequence in HCM patients. In this study, we assessed the electrical activation sequence using three-dimensional ECM, a technique offering non-invasive evaluation under physiological conditions. The normal pattern of ECM in healthy hearts typically shows an initial epicardial breakthrough in the right ventricular anterior para-septal region, followed by activation in an apex-to-base direction, with the last activated region being the postero-basal region of the LV (24). In our study, most HCM patients with a physiological delay in apex-to-base contraction exhibited an ECM activation sequence resembling this normal pattern. However, patients with a loss of this physiological delay, especially those with inverted asynchrony, displayed disruptions in the ECM activation sequence. The first epicardial breakthrough occurred more frequently in the LV postero-basal region, accompanied by an inversion in the activation sequence. Further research is needed to determine whether this pattern is related to an electrical substrate or is the consequence of the specific hypertrophic heart geometry found in the HCM population.

### Pathophysiology

Various factors at different levels of the hypertrophic heart may explain the alterations observed in electrical and contraction sequences:

- At the level of electromechanical coupling and calcium handling: HCM may lead to increased calcium sensitivity (25).
- At the sarcomere level: mutations associated with HCM can enhance functional properties of the myofibers, resulting in a hypercontractile state (26).
- At the cellular level: hypertrophied cardiomyocytes exhibit altered distribution of desmosomes and gap junctions, affecting mechanical tension and electrical impulse transmission (27).
- At the tissue level: the myocardium in HCM often displays architectural disorganization with obliquely or perpendicularly aligned myocytes as well as fibrosis (28).
- At the metabolic level: regional glucose uptake in the hypertrophic heart can be heterogeneous (29).
- At the organ level: microvascular ischemia and local loading conditions can contribute to these alterations. In our study, patients with a loss of ABD exhibited distinct LV geometry, characterized by lower LV volume and higher relative wall thickness, which can influence the intra-ventricular pressure equilibrium.

The complexity of HCM and the multitude of involved parameters may explain why this contraction profile is observed only in a subset of HCM patients.

### Perspectives

The mechanical contraction sequence, allowing for gradual ventricular cavity emptying, may play a role in outflow tract obstruction, especially in those with loss of ABD and an early contraction of the basal septum. Right ventricular apical pacing, used to treat obstruction, may affect contraction at various levels, including electromechanical coupling, electrical activation sequence, and LV contractility. Dual-chamber pacing could be a consideration for a subgroup of obstructive HCM patients exhibiting disruptions in activation and contraction sequences, although clinical benefits in this subgroup remain to be proven.

Our study, which is consistent with previous research, suggests that obstruction is not solely dependent on abnormal electro-mechanical activation sequences, while it may be a contributor. Indeed, there was no difference in contraction patterns between obstructive and non-obstructive HCM patients and no significant correlation between ABD and maximal peak gradient in our study, highlighting that other concomitant factors are at play. The impact of alterations in the mitral valve apparatus, less influenced by the contraction sequence, should be explored further.

### Limitations

Our study has limitations, including a relatively small sample size and operator bias in the analyses. Regarding phase analysis, the Fourier transform allows for the analysis of the entire cardiac cycle rather than just systole, and a time-to-peak approach would be strictly limited to the systolic phase. ECM analysis should be interpreted with caution as the normal activation sequence in hypertrophied hearts has not been well-defined. Additionally, the VVI software, designed for TTE analysis, may not be optimized for CMR analysis.

## Conclusion

A subset of HCM patients exhibit an abnormal LV contraction sequence characterized by the absence of the physiological apex-to-base delay. This phenomenon is associated with specific LV geometry, lower LV volumes, lower native T1 values, distinctive electrical activation patterns observed via electrocardiographic mapping, and possibly early disease. Finally, while these results inform us on HCM's complex pathophysiology, they remain hypothesis-generating and further research is needed to determine their clinical applicability.

## Data Availability

The raw data supporting the conclusions of this article will be made available by the authors, without undue reservation.

## References

[1] Maron BJ Gardin JM Flack JM Gidding SS Kurosaki TT Bild DE. Prevalence of hypertrophic cardiomyopathy in a general population of young adults: echocardiographic analysis of 4111 subjects in the CARDIA study. Circulation. (1995) 92:785–9. 10.1161/01.CIR.92.4.785 7641357

[2] Maron MS Olivotto I Betocchi S Casey SA Lesser JR Losi MA Effect of left ventricular outflow tract obstruction on clinical outcome in hypertrophic cardiomyopathy. N Engl J Med. (2003) 348:295–303. 10.1056/NEJMoa021332 12540642

[3] Vainrib A Massera D Sherrid MV Swistel DG Bamira D Ibrahim H Three-dimensional imaging and dynamic modeling of systolic anterior motion of the mitral valve. J Am Soc Echocardiogr. (2021) 34:89–96. 10.1016/j.echo.2020.08.019 33059963

[4] Luckie M Khattar RS. Systolic anterior motion of the mitral valve—beyond hypertrophic cardiomyopathy. Heart. (2008) 94:1383–5. 10.1136/hrt.2007.122069 18931154

[5] Ommen SR Mital S Burke MA Day SM Deswal A Elliott P 2020 AHA/ACC guideline for the diagnosis and treatment of patients with hypertrophic cardiomyopathy: executive summary: a report of the American college of cardiology/American heart association joint committee on clinical practice guidelines. Circulation. (2020) 142:E533–57. 10.1161/CIR.0000000000000938 33215938

[6] Arbelo E Protonotarios A Gimeno JR Arbustini E Barriales-Villa R Basso C 2023 ESC guidelines on diagnosis and management of hypertrophic cardiomyopathy: the task force for the diagnosis and management of hypertrophic cardiomyopathy of the European Society of Cardiology (ESC). Eur Heart J Eur Heart J. (2023) 44:3503–626. 10.1093/eurheartj/ehad194 37622657

[7] Moura B Aimo A Al-Mohammad A Keramida K Gal TB Dorbala S Diagnosis and management of patients with left ventricular hypertrophy: role of multimodality cardiac imaging. A scientific statement of the heart failure association of the ESC. Eur J Heart Fail Eur. (2023) 25:1493–506. 10.1002/ejhf.2997 37581253

[8] Fananapazir L Cannon RO Tripodi D Panza JA. Impact of dual-chamber permanent pacing in patients with obstructive hypertrophic cardiomyopathy with symptoms refractory to verapamil and beta-adrenergic blocker therapy. Circulation. (1992) 85:2149–61. 10.1161/01.CIR.85.6.2149 1350522

[9] Fananapazir L Epstein ND Curiel RV Panza JA Tripodi D McAreavey D. Long-term results of dual-chamber (DDD) pacing in obstructive hypertrophic cardiomyopathy. Evidence for progressive symptomatic and hemodynamic improvement and reduction of left ventricular hypertrophy. Circulation. (1994) 90:2731–42. 10.1161/01.CIR.90.6.2731 7994815

[10] Nishimura RA Trusty JM Hayes DL Ilstrup DM Larson DR Hayes SN Dual-chamber pacing for hypertrophic cardiomyopathy: a randomized, double-blind, crossover trial. J Am Coll Cardiol. (1997) 29:435–41. 10.1016/S0735-1097(96)00473-1 9015001

[11] Maron BJ Nishimura RA McKenna WJ Rakowski H Josephson ME Kieval RS. Assessment of permanent dual-chamber pacing as a treatment for drug-refractory symptomatic patients with obstructive hypertrophic cardiomyopathy. Circulation. (1999) 99:2927–33. 10.1161/01.CIR.99.22.2927 10359738

[12] Kappenberger L Linde C Daubert C McKenna W Meisel E Sadoul N Pacing in hypertrophic obstructive cardiomyopathy: a randomized crossover study. Eur Heart J. (1997) 18:1249–56. 10.1093/oxfordjournals.eurheartj.a015435 9458416

[13] Galve E Sambola A Saldaña G Quispe I Nieto E Diaz A Late benefits of dual-chamber pacing in obstructive hypertrophic cardiomyopathy: a 10-year follow-up study. Heart. (2010) 96:352–6. 10.1136/hrt.2008.158915 19482844

[14] Lucon A Palud L Pavin D Donal E Behar N Leclercq C Very late effects of dual chamber pacing therapy for obstructive hypertrophic cardiomyopathy. Arch Cardiovasc Dis. (2013) 106:373–81. 10.1016/j.acvd.2013.04.003 23806305

[15] Sengupta PP Khandheria BK Korinek J Wang J Jahangir A Seward JB Apex-to-base dispersion in regional timing of left ventricular shortening and lengthening. J Am Coll Cardiol. (2006) 47:163–72. 10.1016/j.jacc.2005.08.073 16386681

[16] Duchateau J Cornolle C Peyrou J Ritter P Pillois X Réant P Abnormal left ventricular contraction sequence in hypertrophic cardiomyopathy patients: first description of hypersynchrony and invert synchrony. Ultrasound Med Biol. (2015) 41:1632–9. 10.1016/j.ultrasmedbio.2015.01.027 25747939

[17] O’Mahony C Jichi F Pavlou M Monserrat L Anastasakis A Rapezzi C A novel clinical risk prediction model for sudden cardiac death in hypertrophic cardiomyopathy (HCM risk-SCD). Eur Heart J. (2014) 35:2010–20. 10.1093/eurheartj/eht439 24126876

[18] Maron BJ Gottdiener JS Epstein SE. Patterns and significance of distribution of left ventricular hypertrophy in hypertrophic cardiomyopathy. A wide angle, two dimensional echocardiographic study of 125 patients. Am J Cardiol. (1981) 48:418–28. 10.1016/0002-9149(81)90068-0 7196689

[19] Sicari R Nihoyannopoulos P Evangelista A Kasprzak J Lancellotti P Poldermans D Stress echocardiography expert consensus statement: european association of echocardiography (EAE) (a registered branch of the ESC). Eur J Echocardiogr. (2008) 9:415–37. 10.1093/ejechocard/jen175 18579481

[20] Mongeon F-P Jerosch-Herold M Coelho-Filho OR Blankstein R Falk RH Kwong RY. Quantification of extracellular matrix expansion by CMR in infiltrative heart disease. JACC Cardiovasc Imaging. (2012) 5:897–907. 10.1016/j.jcmg.2012.04.006 22974802 PMC3954504

[21] Rudy Y. Noninvasive electrocardiographic imaging of arrhythmogenic substrates in humans. Circ Res. (2013) 112:863–74. 10.1161/CIRCRESAHA.112.279315 23449548 PMC3596167

[22] Serri K Reant P Lafitte M Berhouet M Bouffos VL Roudaut R Global and regional myocardial function quantification by two-dimensional strain: application in hypertrophic cardiomyopathy. J Am Coll Cardiol. (2006) 47:1175–81. 10.1016/j.jacc.2005.10.061 16545649

[23] Stokke TM Hasselberg NE Smedsrud MK Sarvari SI Haugaa KH Smiseth OA Geometry as a confounder when assessing ventricular systolic function: comparison between ejection fraction and strain. J Am Coll Cardiol. (2017) 70:942–54. 10.1016/j.jacc.2017.06.046 28818204

[24] Ramanathan C Jia P Ghanem R Ryu K Rudy Y. Activation and repolarization of the normal human heart under complete physiological conditions. Proc Natl Acad Sci. (2006) 103:6309–14. 10.1073/pnas.0601533103 16606830 PMC1458874

[25] Michele DE Metzger JM. Physiological consequences of tropomyosin mutations associated with cardiac and skeletal myopathies. J Mol Med. (2000) 78:543–53. 10.1007/s001090000161 11199327

[26] Yamashita H Tyska MJ Warshaw DM Lowey S Trybus KM. Functional consequences of mutations in the smooth muscle myosin heavy chain at sites implicated in familial hypertrophic cardiomyopathy. J Biol Chem. (2000) 275:28045–52. 10.1074/jbc.M005485200 10882745

[27] Sepp R Severs NJ Gourdie RG. Altered patterns of cardiac intercellular junction distribution in hypertrophic cardiomyopathy. Heart. (1996) 76:412–7. 10.1136/hrt.76.5.412 8944586 PMC484572

[28] Nam BD Kim SM Jung HN Kim Y Choe YH. Comparison of quantitative imaging parameters using cardiovascular magnetic resonance between cardiac amyloidosis and hypertrophic cardiomyopathy: inversion time scout versus T1 mapping. Int J Cardiovasc Imaging. (2018) 34:1769–77. 10.1007/s10554-018-1385-2 29846837

[29] Perrone-Filardi P Bacharach SL Dilsizian V Panza JA Maurea S Bonow RO. Regional systolic function, myocardial blood flow and glucose uptake at rest in hypertrophic cardiomyopathy. Am J Cardiol Elsevier. (1993) 72:199–204. 10.1016/0002-9149(93)90160-E 8328384

